# Traumatic Distal Ulnar Artery Thrombosis

**DOI:** 10.1155/2014/983160

**Published:** 2014-09-03

**Authors:** Ahmet A. Karaarslan, Ahmet Karakaşlı, Aslan Mayda, Tolga Karcı, Hakan Aycan, Şenol Kobak

**Affiliations:** ^1^Orthopedics and Traumatology Department, Şifa University Faculty of Medicine, 35100 İzmir, Turkey; ^2^Şifa University Bornova Health Application and Research Center, Kazım Dirik Mahallesi, Sanayi Cadde No. 7, Bornova, 35100 İzmir, Turkey; ^3^Orthopedics and Traumatology Department, Dokuz Eylül University Faculty of Medicine, 35340 İzmir, Turkey; ^4^Rheumatology Department, Şifa University Faculty of Medicine, 35100 İzmir, Turkey

## Abstract

This paper is about a posttraumatic distal ulnar artery thrombosis case that has occurred after a single blunt trauma. The ulnar artery thrombosis because of chronic trauma is a frequent condition (hypothenar hammer syndrome) but an ulnar artery thrombosis because of a single direct blunt trauma is rare. Our patient who has been affected by a single blunt trauma to his hand and developed ulnar artery thrombosis has been treated by resection of the thrombosed ulnar artery segment. This report shows that a single blunt trauma can cause distal ulnar artery thrombosis in the hand and it can be treated merely by thrombosed segment resection in suitable cases.

## 1. Introduction

Hypothenar hammer syndrome is usually seen after repetitive trauma in clinics [1–4]. Guyon canal is the place in which the ulnar artery is vulnerable to mechanical forces that entrap the artery near the hook of hamatum. Traumatic thrombosis of the ulnar artery in the hand after a single acute trauma is rare [5].

## 2. Case Report

**Figure 1:**
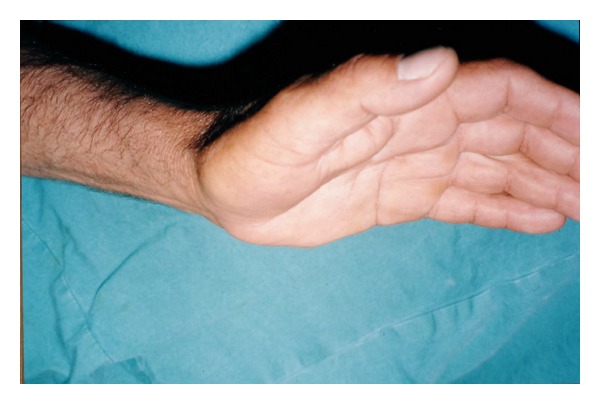
Preoperative appearance of distal ulnar artery thrombosis.

**Figure 2:**
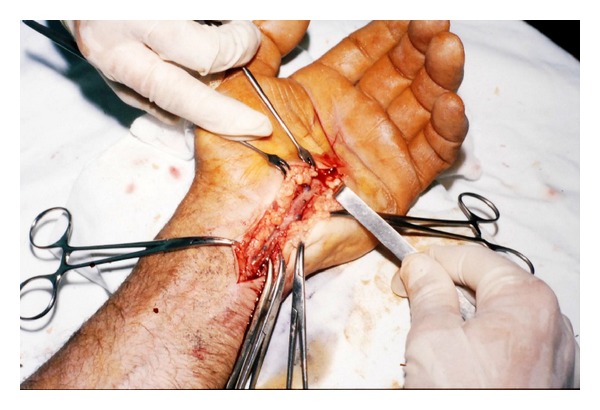
The intraoperative appearance of distal ulnar artery thrombosis.

55-year-old male patient had a blunt trauma on his hypothenar region of the left hand palm by popping out of the car rim while helping to change the car wheel. The patient had no repetitive hand working and minor traumas by his occupational condition. He had pain and swelling on the left hand for four months. On physical examination, a palpably tender, stiff, painful swelling 32 mm in diameter was found to elevate the skin 5 mm high (Figure 1). The Allen test showed no decreased circulation on the ulnar artery area. There were no symptoms of ulnar nerve entrapment preoperatively. Discussing the outcomes and complications of vein graft interposition or distal ulnar artery segment excision with the patient, the patient declared that he does not want reoperations if possible, so we made the decision to excise the artery segment to avoid complications like reoperation. On 10/06/2006, under axillary block anesthesia and pneumatic tourniquet control, the approach was made with a wide zigzag incision on his hypothenar region. With the incision of the ulnar artery segment, we saw that the distal 3,5 cm of the ulnar artery was thrombosed with vena comitantes till the ulnar side of the superficial palmar arch (Figure 2). Ulnar nerve was decompressed. The excision of the thrombosed ulnar artery segment was performed. Intraoperatively, the pneumatic tourniquet was released. The Allen test was performed. The circulation of the fourth and the fifth finger was good. Hypothenar reactive swelling and tenderness disappeared postoperatively, and there were no complaints on the five-year follow-up period. There was no pathology related to the hand functions.

## 3. Discussion

In arterial thrombosis cases, treatment methods like vasodilators, stellate ganglion blockade, cervicodorsal sympathectomy, and chemical thrombolytics are tried [4, 5]. When conservative methods fail, the recommended and usually applied methods are thrombosed artery resection with reversed interpositional vein grafting or just the resection of the thrombosed arterial segment when there is no digital ischaemia [4, 6]. The advantages of surgery are removal of source of microembolism, removal of painful mass, ulnar nerve decompression, and local periarterial sympathectomy [3]. It is concluded that there is not one way for the treatment. The treatment depends on the vascular and general condition of each patient [7].

Zweig et al. [8] performed excision of the thrombosed portion of the distal ulnar artery with ligation of the nonoccluded proximal and distal ends in ten patients. They reported disappearance of the sign and symptoms of distal ulnar artery thrombosis.

Arterial reconstruction of the distal ulnar artery thrombosis using reverse interpositional vein grafting was recommended [4, 6, 9, 10]. A distal ulnar artery venous graft pseudoaneurysm that developed after a hyperextension trauma was reported [11]. It was reported that patients had symptomatic relief even if interpositional vein graft occluded because of local sympathectomy caused by resecting the thrombosed artery [9].

The resection of the thrombosed arterial segment without vein grafting is a suitable option for patients who will continue to use the hand as a hammer or have coagulation deficiency [7]. In our case, to avoid the risk of reoperation, we preferred the resection of the thrombosed arterial segment.

This report highlights that a single isolated blunt trauma can cause distal ulnar artery thrombosis and that it can be treated effectively by resection of the thrombosed distal ulnar arterial segment.
